# Creating a stem cell niche in the inner ear using self-assembling peptide amphiphiles

**DOI:** 10.1371/journal.pone.0190150

**Published:** 2017-12-28

**Authors:** Akihiro J. Matsuoka, Zafar A. Sayed, Nicholas Stephanopoulos, Eric J. Berns, Anil R. Wadhwani, Zachery D. Morrissey, Duncan M. Chadly, Shun Kobayashi, Alexandra N. Edelbrock, Tomoji Mashimo, Charles A. Miller, Tammy L. McGuire, Samuel I. Stupp, John A. Kessler

**Affiliations:** 1 Department of Otolaryngology and Head and Neck Surgery, Feinberg School of Medicine, Northwestern University, Chicago, Illinois, United States of America; 2 Department of Communication Sciences and Disorders, Northwestern University, Evanston, Illinois, United States of America; 3 Hugh Knowles Center for Hearing Research, Northwestern University, Evanston, Illinois, United States of America; 4 Simpson Querrey Institute for BioNanotechnology, Northwestern University, Chicago, Illinois, United States of America; 5 Department of Biomedical Engineering, Northwestern University, Evanston, Illinois, United States of America; 6 Department of Neurology, Feinberg School of Medicine, Northwestern University, Chicago, Illinois, United States of America; 7 The Institute of Experimental Animal Sciences, Graduate School of Medicine, Osaka University, Suita, Osaka, Japan; 8 Department of Medicine, Feinberg School of Medicine, Northwestern University, Chicago, Illinois, United States of America; 9 Department of Chemistry, Northwestern University, Evanston, Illinois, United States of America; 10 Department of Materials Science and Engineering, Northwestern University, Evanston, Illinois, United States of America; University of Kansas Medical Center, UNITED STATES

## Abstract

The use of human embryonic stem cells (hESCs) for regeneration of the spiral ganglion will require techniques for promoting otic neuronal progenitor (ONP) differentiation, anchoring of cells to anatomically appropriate and specific niches, and long-term cell survival after transplantation. In this study, we used self-assembling peptide amphiphile (PA) molecules that display an IKVAV epitope (IKVAV-PA) to create a niche for hESC-derived ONPs that supported neuronal differentiation and survival both in vitro and in vivo after transplantation into rodent inner ears. A feature of the IKVAV-PA gel is its ability to form organized nanofibers that promote directed neurite growth. Culture of hESC-derived ONPs in IKVAV-PA gels did not alter cell proliferation or viability. However, the presence of IKVAV-PA gels increased the number of cells expressing the neuronal marker beta-III tubulin and improved neurite extension. The self-assembly properties of the IKVAV-PA gel allowed it to be injected as a liquid into the inner ear to create a biophysical niche for transplanted cells after gelation in vivo. Injection of ONPs combined with IKVAV-PA into the modiolus of X-SCID rats increased survival and localization of the cells around the injection site compared to controls. Human cadaveric temporal bone studies demonstrated the technical feasibility of a transmastoid surgical approach for clinical intracochlear injection of the IKVAV-PA/ONP combination. Combining stem cell transplantation with injection of self-assembling PA gels to create a supportive niche may improve clinical approaches to spiral ganglion regeneration.

## Introduction

The use of cochlear implants (CIs) is the standard of care for patients with severe-to-profound sensorineural hearing loss (SNHL) [1], though users frequently note poor speech perception in noisy environments and often find it challenging to appreciate music [2]. One promising treatment strategy involves the repopulation of spiral ganglion neurons (SGNs) in the cochlea, which undergo irreversible retrograde trans-synaptic degeneration in this patient population [3]. Despite recent encouraging progress in regenerating SGNs in animal models by transplanting cells derived from human embryonic stem cells (hESCs) into the inner ear [4,5], clinical translation requires increasing the efficiency of otic neural progenitor cell (ONP) production, neuronal differentiation, preferential placement of ONPs, and long-term in vivo survival. Chen et al. encouragingly reported restored auditory brainstem responses after transplanting hESC-derived ONPs [4]. However, other studies using murine stem cells found poor stem cell survival (< 1%) one week after in vivo transplantation [5–7]. In a recent study, we described a protocol for controlled and efficient creation of hESC-derived ONP populations [8]. Here, we focus on a subsequent step: creating a supportive extracellular niche in the inner ear in vivo that supports survival and adequate neuronal differentiation of transplanted hESC-derived ONPs.

Stem cells normally reside in a tissue microenvironment, or niche, that regulates their proliferation, differentiation, and survival [9,10]. Transplantation of stem cells into an inhospitable microenvironment limits engraftment and survival [11,12], indicating the need for techniques for creating supportive cell niches. Although the precise constituents of the normal microenvironment in the inner ear are still unknown, it may be possible to construct a functional niche in the inner ear using self-assembling peptide amphiphiles (PAs) designed to provide local signals that promote both survival and neuronal differentiation of transplanted stem cells [11,12]. These PAs self-organize into oriented nanofibers 6–12 nm in diameter and several microns in length. Hydrogen bonding between amino acids in adjacent molecules then drives fiber formation and gelation initiated by contact with physiological concentrations of divalent cations, such as Ca^2+^ [13–15]. This design feature allows the material to remain liquid during handling and delivery, with eventual transformation into an oriented scaffold following delivery. Moreover, because of their predominantly aqueous composition (>99% by weight), these liquid crystalline gels provide adequate space for long-term cellular growth [11].

Prior work has demonstrated the ability of solutions of IKVAV-PA to form gels when injected into neural tissue [11,16–19]. These gels have provided a three-dimensional (3-D) guide for murine neural progenitor migration and outgrowth mediated by the neurite-promoting laminin epitope, IKVAV [11]. A mixture of PA gels presenting either IKVAV or RGD, another peptide signaling epitope, enhanced axonal regeneration of peripheral nerves and Schwann cell immigration compared to control PA gel with no epitope [20]. Various IKVAV-PA gels were also recently shown to increase the probability of neurite attachment from spiral ganglion explants in vitro as compared to their non-functionalized counterparts, suggesting suitability for use in the inner ear [21]. Additionally, coating the auditory nerve with IKVAV-PA gels potentially may promote neurite growth that bridges the Obersteiner-Redlich zone (ORZ)—the Schwann-glial cell junction central to the cochlea—overcoming growth inhibition observed at this junction [22,23]. A recent study demonstrated that hand-fabricated macroscopically aligned gels containing encapsulated neurons and bioactive PA fibers can orient neurite growth and control the direction of cell migration [18].

Inner-ear stem cell transplantation therapy has been limited conventionally to injection of dissociated suspended neuronal progenitors or mature neurons, which typically results in poor survival [5,6,24–26]. With other organs (including the brain) and neuron types, this approach has yielded similar disappointing outcomes [27,28]. Our study therefore focused on implantation of late-stage ONPs, as opposed to injection of fully developed SGN-like neurons. We hypothesized that using IKVAV-PA gels to create robust stem cell niches in the inner ear and internal auditory canal (IAC) would promote survival and neuronal differentiation of transplanted hESC–derived ONPs. Accordingly, we first examined the effects of IKVAV-PA gels on hESC-derived ONPs in vitro and then tested the PA gels in vivo using X-SCID rats (severely combined immunodeficient animals suitable hosts for xenogeneic stem-cell transplantation) [29]. Although autologous transplantation of induced pluripotent stem cells (iPSCs) is the ideal scenario for bypassing immunorejection after transplantation, our study focused on cells derived from hESCs rather than autologous rodent iPSCs since studies of human cells will likely translate more readily to a clinical setting. Finally, we demonstrated the technical feasibility of future clinical application using human cadaveric temporal bones. Our findings suggest that combining stem cell transplantation with injection of self-assembling PA gels to create a supportive cell niche may improve the success of stem cell-based approaches to auditory nerve regeneration.

## Materials and methods

### PA gel preparation

IKVAV PA gels (palmitoyl-VVAAEEEEGIKVAV-COOH) were synthesized as outlined by McClendon et al [12]. A PA gel with a scrambled IKVAV sequence (VVIAK: valine, valine, isoleucine, alanine, and valine) was also created as a control. PAs were synthesized using standard fluorenylmethoxycarbonyl (FMOC) solid-phase peptide synthesis and purified by preparative-scale reverse-phase HPLC with water and acetonitrile containing 0.1% NH_4_OH. Solutions of PAs, composed of 0.5% E2 PA (palmitoyl-VVAAEE-NH_2_) and either 0.125% IKVAV-PA or VVIAK-PA in 150 mM NaCl and 3 mM KCl (pH 7.4), were thermally annealed at 80°C for 30 minutes and slowly cooled to room temperature overnight to induce formation of aligned bundles of nanofibers suitable for shear-induced alignment [30]. Cells were mixed (as described below) with the PA solutions after the annealing and cooling steps. A PA covalently conjugated to the fluorophore TAMRA (Ex/Em 565/580 nm) was added at 0.001 wt % in PA gels for fluorescence imaging for the injection into human cadaveric temporal bones. 3-D visualization of the IKVAV molecule was constructed using the open-source MolView v2.4 software (molview.org).

### Human ESC cultures

Frozen undifferentiated H1, H7, and H9 hESCs (passages 25–35) (WiCell Research Institute, Madison, Wisconsin, USA) were cultured in hESC culture medium as previously described (also see S1 Supporting Information) [8,31]. Maintenance was performed through daily media changes and passaging using Gentle Cell Dissociation Reagent (STEMCELL Technologies, Cambridge, Massachusetts, USA). Pluripotency was maintained via removal of differentiated colonies by standard picking and manual passage techniques, and verified prior to ONP differentiation by magnetic activated cell sorting for TRA-1-60^+^ cells (Miltenyi Biotec, Bergisch Gladbach, Germany). In human cadaveric temporal bone experiments, the resultant cell pellet was washed with phosphate-buffered saline (PBS) prior to staining for TRA-1-81 (undifferentiated hESC marker) [32].

### Generation of hESC-derived ONPs

**Fig 1:**
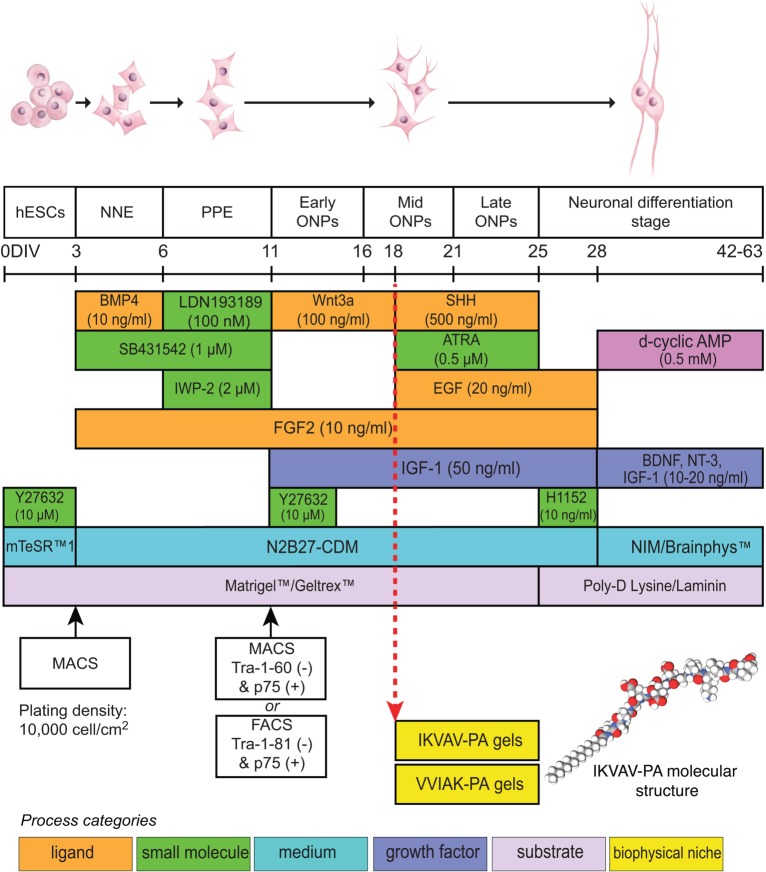
Schematic summary of the protocol and timeline for deriving the SGN lineage from undifferentiated hESCs. DIV: day in vitro; NNE: nonneuronal ectoderm; PPE: preplacodal ectoderm; ONP: otic neural progenitor; BMP4: bone morphogenetic protein 4; SHH: Sonic hedgehog; ATRA: all-trans retinoic acid; EGF: epidermal growth factor; BDNF: brain-derived neurotrophic factor; NT-3: neurotrophin 3; IGF-1: insulin-like growth factor 1; FGF2: fibroblast growth factor 2; E8: Essential 8^™^ medium; N2B27-CDM: chemically defined medium containing N2 and B27 supplements; MACS: magnetic-activated cell sorting; FACS: fluorescence-activated cell sorting; p75: low-affinity neurotrophin receptor (p75^NTR^); IKVAV-PA: IKVAV-containing peptide amphiphile; VVIAK-PA: VVIAK-containing peptide amphiphile. Protocol adapted and modified from Matsuoka et al. [8].

Differentiation of hESCs was performed following the protocol recently published by Matsuoka et al. [8]. Undifferentiated hESCs were passaged from the feeder layer and cultured in a 100-mm dish coated with Matrigel^™^ (BD Pharmingen, San Jose, California, USA) using Essential 8^™^ Medium (Life Technologies, Grand Island, New York, USA) or mTeSR^™^1 (STEMCELL Technologies, Cambridge, Massachusetts, USA). In some cases, Geltrex^™^ (Thermo Fisher Scientific, Waltham, Massachusetts, USA) was substituted for Matrigel^™^. The authors noted little difference between the two coatings in terms of ONP generation. Human ESCs were allowed to proliferate for 2 days prior to adding a chemically defined medium containing N2 and B27 supplements (N2B27-CDM). Ligands and growth factors were added in stepwise fashion to promote hESC differentiation to ONPs as shown in Fig 1, adapted from Matsuoka et al. and previously described [8]. Early-stage ONPs are defined as preplacodal ectoderm (PPE)-like cells treated with 100 ng mL^-1^ human Wnt3a, 10 ng mL^-1^ human fibroblast growth factor 2 (FGF2), and 50 ng mL^-1^ insulin-like growth factor (1GF-1) (W/F/I) for 5 days. Mid-stage ONPs are defined as PPE-like cells treated with W/F/I for two additional days, followed by treatment with 500 ng mL^-1^ Sonic hedgehog (SHH), 0.5 μM all-trans retinoic acid (ATRA), 10 ng mL^-1^ FGF2, 20 ng mL^-1^ EGF, and 50 ng mL^-1^ IGF-1 (S/R/E/F/I) for 3 days. Late-stage ONPs are defined as mid-stage ONPs treated with S/R/E/F/I for 4 additional days (Fig 1). In a monolayer (2-D matrix) culture environment, mid-stage ONPs were neuronally differentiated into late-stage ONPs on Matrigel™. Alternatively, IKVAV-PA gels and VVIAK-PA gels were used to assess neuronal differentiation into late-stage ONPs within 3-D matrices. Mid-stage ONPs were seeded onto a 24-well plate at densities of 10,000–25,000 cells cm^-2^ (poly-ornithine/laminin) or, for the PA gels, cell suspensions were mixed with PA solution (after the heating and cooling steps) at a 1:2 ratio (75 μL total volume per well, 5,000 cells μL^-1^). Cells were allowed to grow for 7 additional days.

### Immunocytochemistry, image acquisition, and quantification of cells

Immunocytochemistry was performed using previously described standard techniques [11,18]. Briefly, cells were fixed with 4% paraformaldehyde solution, permeabilized with a nonionic surfactant, and blocked using bovine serum albumin or fetal bovine serum for gels. Cells were then incubated sequentially in primary and fluorescent secondary antibody solutions to allow visualization of targeted proteins by fluorescence microscopy (full details, antibody sources, and concentrations included in S1 Supporting information). Secondary antibody-only controls were performed by omitting the primary antibody during incubation (S1 Fig). For positive controls for immunofluorescence staining of ONPs refer to previous work [8]. Fluorescence imaging was performed on a Zeiss UV-LSM 510 META or a Nikon A1/C2 confocal microscope. Sequential scanning of channels was performed to prevent false-positive co-localization. ImageJ 1.51e 5 [33] was used to quantify images.

### Analysis of neurite-bearing cells and growth

To confirm the neuronal origin of the processes extending from mid-stage ONPs treated with S/R/E/F/I and from late-stage ONPs with neuronal differentiation, immunostaining with an antibody against the neuron-specific marker β-III tubulin was used to visualize neurite-bearing cells and quantify their number and length. Images of randomly selected 20 areas of cells on Matrigel™ (control), within VVIAK-PA gels, and within IKVAV-PA gels were obtained at 40× magnification, digitized, and analyzed using ImageJ 1.51e 5 [33]. Neurite-bearing cells were quantified by counting the number of neurons in a field with neurite lengths at least twice the diameter of the cell body. The ratio of neurite-bearing cells to total number of cells was computed for each condition. Neurite length is determined by manually tracing the length of the longest neurite on randomly selected 20 late-stage ONPs using the NIH Image J software 1.51e 5 [33].

### Live/dead cell viability assay and EdU cell proliferation assay

In vitro cell viability was assessed using a LIVE/DEAD Viability/Cytotoxicity kit (Life Technologies, Carlsbad, California, USA) with standard techniques. Briefly, cells were stained with both ethidium bromide and calcein as markers for abnormal cell membrane permeability (indicating cell death) and intracellular esterase activity (indicating healthy cell function), respectively. Click-iT^®^ EdU DNA incorporation assays (Invitrogen, Carlsbad, California, USA) were performed to confirm in vitro cell proliferation using standard techniques. For these assays, cells were fixed and permeabilized as for immunocytochemistry, stained with EdU reagent, and analyzed by fluorescence microscopy.

### Generation of EGFP^+^ hESCs using a CRISPR-Cas9 system

EGFP was knocked into the AAVS1 safe harbor locus of H7 strain hESCs using a CRISPR-Cas9 based strategy as previously described with modifications [34,35]. Briefly, hESC line H7 was cultured in mTeSR^TM^1 medium (STEMCELL Technologies, Cambridge, MA, USA) on multi-well culture plates coated with Matrigel^TM^ (BD Pharmigen, San Jose, California, USA) per manufacturer-provided protocols. Cells were fed daily with fresh media. Spontaneously differentiated cells were removed by regular inspection and manual dissection. Cells were passaged as clumps every 5–6 days with Accutase (EMD Millipore, Kankakee, Illinois, USA) at a split ratio of approximately one to twelve. Cells were pre-incubated with 10 μM ROCK Inhibitor Y-27632 (Stemgent, Cambridge, Massachusetts, USA) for 4 hours and dissociated to single cells with Accutase (EMD Millipore, Kankakee, Illinois, USA). The cell suspension was washed and resuspended in DPBS (Life Technologies, Carlsbad, California, USA) containing 1 μg hCas9 vector (Addgene #41815), 1 μg gRNA_AAVS1-T2 vector (Addgene #41818), and 2 μg AAV-CAGGS-EGFP vector (Addgene #22212). The suspension was transferred to an Amaxa electrode cuvette and nucleofected with a Nucleofector II/2b Device (Lonza, Valparaiso, Indiana, USA) using program B-016. 0.5 mL of pre-warmed mTeSR^TM^1 media was added to the cells, and the mixture was transferred to conical vial containing 2 mL of additional media. The cells were centrifuged and resuspended in mTeSR^TM^1 media supplemented with 10 μM ROCK Inhibitor Y-27632 and plated at a density of 20,000 cell cm^-2^ on Matrigel^TM^-coated plates. Cells were fed daily with fresh mTeSR^TM^1 medium. Cells were selected by supplementing media with 0.5 μg mL^-1^ of puromycin 72 hours after transfection. Individual puromycin-resistant hESC colonies were manually picked and expanded. Genomic DNA was isolated using the DNEasy Blood and Tissue Kit (Qiagen, Hilden, Germany), and on-target insertion of the EGFP cassette was confirmed by PCR using primers with complementarity within the AAVS1 homology arms: Forward primer: 5’-CCCCTTACCTCTCTAGTCTGTGC-3’; Reverse primer: 5’-CTCAGGTTCTGGGAGAGGGTAG-3’. The PPP1R12C locus was PCR-amplified with pfx polymerase (Life Technologies, Carlsbad, California, USA) and indicated primers (IDT, Coralville, IA, USA), and the product was analyzed by agarose-gel electrophoresis. Live cells were visualized by brightfield and epifluorescent microscopy (Nikon, Tokyo, Japan).

Percent EGFP retention after late-stage ONP differentiation was assessed by counting fluorescent and total cells in the same field captured by live cell epifluorescent microscopy (Nikon, Tokyo, Japan). Cell counting was performed manually using the NIH ImageJ 1.51e 5 [33]. Retention of fluorescence at this time point is presumed to follow a binomial distribution for error estimation:

$$\sigma =\sqrt{np(1-p)}$$

(*σ*: standard deviation, *n*: number of cells, *p*: probability that a cell has lost fluorescence after differentiation.)

### RT-qPCR

RNA was extracted from undifferentiated hESCs (H7), hESC (H7)-derived late-stage ONPs, and EGFP^+^ hESC (H7) derived late-stage ONPs using the mirVANA™ miRNA isolation Kit (Life Techonologies, CA, USA). Twelve-point-five ng of RNA was reverse-transcribed with random primers using the iScript™ Reverse Trancription Superkit for RT-qPCR (Bio-Rad Laboratories, Hercules, California, USA) and the following cycling conditions: 25°C for 5 min, 46°C for 20 min, and 95°C for 1 min. The cDNA was amplified by qPCR using following primers (OCT3/4 forward: 5' ATG TGC AAG CTG AAG CCT TT 3'; OCT3/4 reverse: 5' GCT GCG ATC TTG TCT ATG CTC 3'; NANOG forward: 5' AAC TGG CCG AAG AAT AGC AA 3'; NANOG reverse: 5' CAT CCC TGG TGG TAG GAA GA 3'; GAPDH reverse: 5' TGT TCG TCA TGG GTG TGA AC 3'; GAPDH forward: 5' CTA AGC AGT TGG TGG TGC AG 3') on a Bio-Rad CFX384 (Bio-Rad Laboratories, Hercules, CA) with the following cycling conditions: 45 cycles: (95°C for 10 sec, 64°C for 15 sec, 72°C for 20 sec). *C*_*Τ*_ values were measured in two technical replicates and all values represent three biological replicates.

GAPDH was initially to be used as a reference gene for normalization. However, gene expression varied across conditions (S2 Fig). To avoid the introduction of bias, normalization was performed using the NORMA-gene method [36]. A normalization factor is computed for each cell type replicate based on the variation among replicates within the cell type across all target genes measured. The normalization factor is computed using a least square method, minimizing variability in the dataset between replicates. This normalization factor is added to each raw *C*_*Τ*_ value in order to yield a Δ*C*_*Τ*_ value. The relative expression in undifferentiated and differentiated hESCs was then analyzed using the ΔΔ*C*_*Τ*_ method, comparing each Δ*C*_*Τ*_ to the mean Δ*C*_*Τ*_ value for each gene target for undifferentiated hESC samples [37].

### Human ESC-derived ONP transplantation into X-SCID rat cochleae

X-SCID F344-Il2rg^em7kyo^ rats were generated by Dr. Tomoji Mashimo (Graduate School of Medicine, Kyoto University Institute of Laboratory Animals). This strain has severe combined immunodeficiency caused by a 7-bp deletion in exon 2 of the interleukin 2 receptor gamma (Il2rg) gene[29]. The rats were transferred to Northwestern University and housed under specific-pathogen-free conditions. The Institutional Animal Care and Use Committee of Northwestern University Feinberg School of Medicine approved our experiment procedures (IACUC Protocol number: IS00000379), which also met U.S. National Institutes of Health guidelines for animal care and use.

Twelve X-SCID rats (6 each for control and experimental groups), 6–8 weeks of age, were used as stem-cell transplant recipients using sterile techniques. A CRISPR-Cas9 system was used to generate EGFP^+^ hESCs for transplantation into X-SCID rat cochlea. A cochleostomy was performed posterior to the round window niche and a 23-gauge beveled needle was used to fenestrate the scala tympani medial wall for modiolar access. For injection, EGFP^+^ late-stage ONPs were first dissociated with Accutase (EMD Millipore, Kankakee, Illinois, USA) and suspended in PA solution at approximately 5,000 cells μL^-1^. EGFP^+^ late-stage ONPs were also mixed culture medium (DMEM) at 5,000 cells μL^-1^ (control). About 4 μL (2 x 10^4^ cells) were injected into the modiolus using a 10-μL Hamilton syringe (World Precision Instruments, Sarasota, Florida, USA). The rate of the injection was 1 μm min^-1^. At that rate, we avoided damage to inner-ear tissues due to the injection [6,7,26]. The PA soluion (IKVAV-PA gels and VVIAK-PA gels) was injected as a liquid because it is technically not feasible to inject any material that has been already in gelled into the entire scala tympani, especially with the intent of filling the entire structure. Previous studies have already demonstrated that the self-assembly properties of the IKVAV-PA gel allowed for the liquid form of PA gels to establish the gelled state after injection in vivo as long as the local environment (the subarachnoid space) contains divalent cations such as calcium ions (Ca^2+^) [11,16–19]. Perilymph in the scala tympani has a similar ionic composition to CSF in mammals [38]. We have also performed an additional rheological study of the IKVAV-, VVIAK-PA hydrogels after gelation. Most importantly, the frequency sweep showed G’> G” (G’: the storage modulus; G”: the loss modulus) in all cases at all frequencies indicating the material is in a gelled state (see S1 and S2 Supporting Informations and S3 Fig for details). Furthermore, IKVAV nanofiber orientation is determined by tensile forces created during its ejection from the needle. Thus, to promote nanofiber alignment toward the modiolus, the injection needle was slowly withdrawn from the modiolar injection site during injection. Rats were allowed to survive 4 weeks post-implantation to assess surviving EGFP^+^ cells using an anti-EGFP antibody. Details of a similar transplantation surgery in gerbils have been reported elsewhere [6,7,26]. More detail is also available in S1 Supporting information.

Compared to the gerbil surgery, several minor differences were accounted for [39]. The facial nerve in the rat middle ear is in a more superficial, anterior-rostral position than in the gerbil, and is thereby less protected. Care was taken during surgery to avoid injury causing paralysis to the facial nerve, which can adversely affect the animal’s general health. To safely perform the small cochleostomy, a three-flanged hand drill manufactured from quality stainless steel wire was used. Because localized damage to the basilar membrane in the lower basal turn can occur during cochleostomy near the round window, it is important to make the cochleostomy as posterior as possible to reduce the risk of intracochlear trauma.

To quantify EGFP^+^ ONP survival after transplantation, profile counts were generated from an image taken with the confocal and epifluorescent microscopes [40]. Due to the lack of a clear demarcation line segregating EGFP^+^ ONPs in the rat cochlea and the heterogeneous distribution of the EGFP^+^ ONPs, the profile count method was chosen over stereology-based methods. Six cochleae were injected with EGFP^+^ ONPs with IKVAV-PA gels and six cochleae were injected with control cells suspended in DMEM. EGFP^+^ cells and DAPI-stained cells were counted using the ITCN (image-based tool for counting nuclei) plugin for ImageJ [41]. The total profile number was calculated by counting profiles of the EGFP^+^ ONPs on the five most central modiolar sections. The profile number obtained from each of the five sections was multiplied by 4, as every fourth section was kept for analysis. The total profile number of the EGFP^+^ cells was determined in each of the five sections for each XSCID rat in the four anatomic subdivisions of the cochlea: the scala tympani (ST), scala media (SM), scala vestibule (SV), and modiolus (MO). One-factor factorial analysis of variance was used for statistical evaluation of the profile counts, and the significance of the difference in the profile count of transplanted EGFP^+^ ONPs across the compartments of the cochleae was tested using Tukey-Kramer’s test.

### Ex vivo characterization of IKVAV-PA gels after injection into human cadaveric temporal bone

Two formalin-fixed cadaveric temporal bones underwent radical mastoidectomy for improved visualization of the inner ear in December 2013. Specimens were donated anonymously to Northwestern University Feinberg School of Medicine for scientific and educational use. The study fulfilled all the requirements of the Declaration of Helsinki regarding the ethical use of human cadaveric material [42]. Using a high-speed drill with 0.5–1.0 mm diamond burrs, a cochleostomy was created immediately anterior and inferior to the round window niche. A 33-gauge Hamilton needle (Bonaduz, GR, Switzerland) was used to breach the medial wall of the scala tympani adjacent to the modiolus. Next, using a middle cranial fossa approach, the bony covering of the IAC was drilled down to intact dura to access the nerve bundles and provide intracranial visualization. For 12 hr, the two whole-bone specimens (“A” and “B”) were soaked in a 25 mM CaCl_2_ solution with 0.9% normal saline that approximated cerebrospinal fluid and promoted gel solidification after transmodiolar injection. The modiolar access was cannulated using a 25-gauge spinal needle attached to a 1 mL Hamilton syringe. For specimen A, 250 μL (i.e., approximate IAC volume [43]) of TAMRA-tagged IKVAV gel (0.25% E2 PA, 0.0625% IKVAV PA, 0.001% TAMRA PA) with hESC density of 5,000 cells mL^-1^ and dilution ratio of 1:1 was injected into the modiolus. For specimen B, 300 μL of untagged IKVAV gel with TRA-1-81-tagged hESCs in N2B27-CDM was injected. Injections were performed manually over 2 minutes, with care taken to provide a constant fluid flow rate. A subsequent period of ~1 hr provided time for gel solidification. Overlying dura was removed to examine the VCN for red-wavelength fluorescence (excitation of TAMRA at 555 nm, Kodak Image Station In-Vivo F, DataMax v2.20) so as to assess passage of both gel and cellular components. Endoscopic images aligned with the injection pathway were obtained using a rigid 0° 16-mm Gyrus ACMI micro-endoscope (Gyrus ACMI Surgical Endoscopy Division, Southborough, Massachusetts, USA). This endoscope was also used to assist in the injection of ONPs into the modiolus in the cadaveric human bones, as it provided a favorable angle to the modiolus not attainable with a conventional operating microscope. Temporal bone imaging prior to PA gel injection was obtained for comparison to experimental procedures.

### Transmission electron microscopy (TEM) and scanning electron microscopy (SEM)

TEM and SEM were performed using standard techniques. For TEM, tissues were sectioned and stored in paraformaldehyde with CaCl_2_, then dehydrated in an ethanol series. Specimens were post-fixed with osmium tetroxide and stained with uranyl acetate, then embedded into an epoxy resin and sectioned on a microtome. Sections were again stained with uranyl acetate and lead citrate, further sectioned to a thickness of 70 nm, and imaged using a FEI Tecnai Spirit G2 electron microscope. SEM samples were prepared by dehydration through an ethanol series and critical point drying process followed by osmium coating using an osmium plasma coater. SEM images were taken on a Hitachi S-4800 field emission scanning electron microscope.

### Statistical analysis

Statistical analysis was performed with one-way ANOVA (with Tukey-Kramer’s post hoc test to identify significant differences between means while controlling the family-wise error rate) or with a two-tailed, unpaired Student's *t*-test. Equal population variance was not assumed in *t*-tests in order to perform more stringent statistical analysis, but was assumed during ANOVA. A free statistical software package, R (version 3.2.5), was used to perform these tests [44]. Mean values are typically expressed as mean +/- standard error. A significant *p*-value is indicative of a significant difference where the probability is less than (*p* < 0.05*), 0.01 (*p* < 0.01**), and 0.001 (*p* < 0.001***).

## Results

### Differentiation of ONPs in vitro

#### Immunocytochemistry

**Fig 2:**
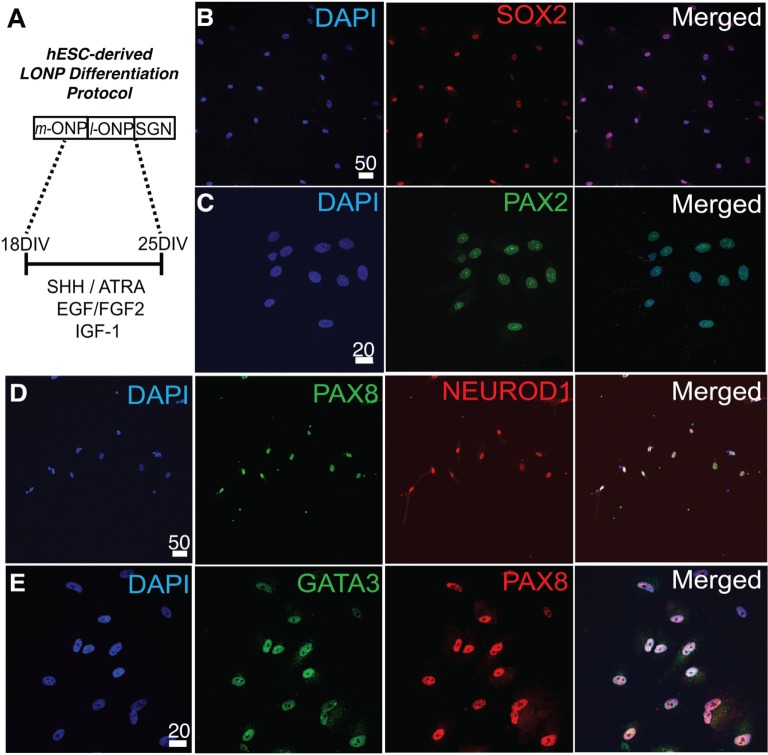
Immunocytochemical assessment of differentiation of mid-stage hESC-derived ONPs into late-stage ONPs. **(A)**: An experimental paradigm. Immunocytochemistry of late-stage ONPs derived from H1, H7, and H9 undifferentiated ESCs for SOX2 **(B)**, PAX2 **(C)**, PAX8/NEUROD1 **(D)**, and GATA3/PAX8 **(E)**. Human hESC cell lines used were H7 (C), H9 (B), and H1 (D, E). Scale bars: 50 μm (B and D) and 20 μm (C and E). m-ONP: mid-stage ONPs; l-ONP: late-stage ONPs; SGN: spiral ganglion neurons; S/R/E/F/I: SHH/ATRA/EGF/FGF2/IGF-1.

The sequential differentiation of hESCs into mid-stage ONPs used the protocol of Matsuoka et al. [8] is summarized and modified in Fig 1 to include details regarding the IKVAV-PA scaffold and the controlled scaffold. ONPs were then cultured on a 2-D matrix (Matrigel™), embedded in 3-D scrambled control PA gels (VVIAK), or experimental PA gels (IKVAV). Derived mid-ONPs plated on Matrigel™ and treated with S/R/E/F/I for seven days (Fig 2A) expressed neuronal progenitor markers (NEUROD1 [45] and SOX2 [46]) and otic markers (PAX2 [47]), indicating that the cells belong to the ONP lineage (Fig 2B, 2C and 2D) [8]. In addition, cells were positive for GATA3 [48] and PAX8 [8,49] showing that they are also at the later stage of ONPs (Fig 2D and 2E). Furthermore, these cells did not express NANOG, SOX10, GFAP, PAX6, or E-cadherin, distinguishing them from hair cells, pluripotent cells, and progenitors fated for neural crest, astroglial, or central neuron lineages [50–53]. A more complete immunocytochemical characterization of late-stage ONPs using H1, H7, and H9 hESCs is provided in Matsuoka et al. [8].

**Fig 3:**
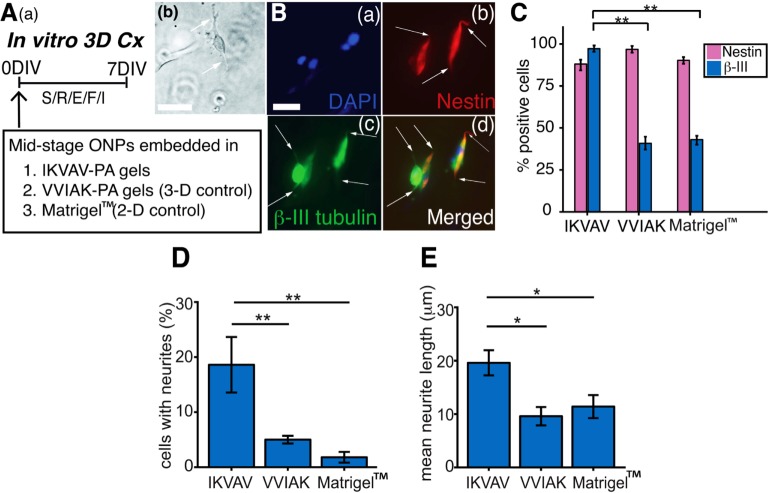
Neuronal differentiation of hESC-derived late-stage ONPs in IKVAV and VVIAK 3D matrices. **(Aa)**: An experimental paradigm on neuronal differentiation of hESC-derived mid-stage ONPs treated with S/R/E/F/I in IKVAV & VVIAK PA gels (3-D) and Matrigel™ (2-D) for seven days. DIV: days in vitro. **(Ab)**: A photomicrograph of phase-contrasted image of a mid ONP treated with S/R/E/F/I in IKVAV-PA gels. A White arrow indicates a neurite. Scale = 20 μm. **(B)**: Immunocytochemistry of nestin and β-III tubulin for hESC-derived mid-ONPs treated with S/R/E/F/I in IKVAV-PA gels. DAPI stain is shown in blue. A white arrow indicates neurites. Scale bar: 20 μm. **(C)**: Quantification of nestin and β-III tubulin-immunopositive cells on derived mid-stage ONPs treated with S/R/E/F/I in IKVAV-PA gels, VVIAK-PA gels, and Matrigel™ for 7 days. β-III: β-III tubulin. (**D**): Quantification of neurite-bearing cells on derived mid-stage ONPs treated with S/R/E/F/I. in IKVAV-PA gels, VVIAK-PA gels, and Matrigel™. (**E**): Quantification of average neurite length on S/R/E/F/I-treated hESC-derived mid-stage ONPs. ** *p* < 0.01, * *p* < 0.05 by one-way ANOVA with Tukey-Kramer’s post-hoc test.

Fig 3A(a) diagrams how IKVAV-PA gels were used to create 3-D matrices in vitro. VVIAK-PA gels, which form the same 3-D structure but do not activate Integrin signaling, was used a control [11]. Matrigel^™^ was also used to compare differentiation to our previously established 2-D culture system [8]. Fig 3A(b) shows a phase contrast photomicrograph of a hESC-derived mid-stage ONP treated with S/R/E/F/I for seven days in IKVAV-PA gels. The neurite-bearing cell displays the typical bipolar morphology of the otic neuronal lineage. Immunocytochemical characterization of late-stage ONPs embedded into IKVAV-PA gels is shown in Fig 3B; these bipolar cells also have extended neurites (white arrows in Fig 3B(b), 3B(c) and 3B(d)). Quantification of β-III tubulin expression and of neurite extension demonstrate the advantages of the IKVAV-PA matrix. With Matrigel^™^, only 42.4% ± 3.40 of these late-stage ONPs expressed the neuronal marker, β-III tubulin [54] (Fig 3C). Cells embedded in “scrambled” (VVIAK-PA) gels generated late-stage ONPs with strong nestin positivity (Fig 3C); however, only 39.6% ± 4.17 of cells expressed β -III tubulin (Fig 3C). In contrast, almost all cells in IKVAV-PA gels expressed strong positivity for both β -III tubulin (97.2% ± 1.83) (p< 0.01) and nestin (88.0% ± 3.74) (Fig 3C), suggesting that this matrix enhanced neuronal differentiation. The percentage of cells extending neurites (Fig 3D) was significantly higher in IKVAV-PA gels than in VVIAK-PA (*p* < 0.01) or Matrigel™ (*p* < 0.01) matrices (18.6% ± 5.05 for IKVAV-PA gels; 5.00% ± 0.71 for VVIAK-PA gels, and 1.80% ± 0.97 for Matrigel™). The average length of neurites (Fig 3E) was also about twice as long in IKVAV-PA gels than in the two controls (19.6 μm ± 2.36 for IKVAV-PA; 9.60 μm ± 1.72 for VVIAK-PA (*p* < 0.05); and 11.4 μm ± 2.16 for Matrigel™ (*p* < 0.05)). The combined enhancement in β -III tubulin expression, the number of neurite bearing cells, and also in neurite length indicate that the IKVAV-PA gels promote neuronal differentiation of hESC-derived ONPs.

#### Live/dead assay

Live/dead assays were performed to evaluate the viability of hESC-derived late-stage ONPs cultured on the three matrices. More than 75% of the cells survived in all conditions and there were no significant differences in the survival of cells cultured on the different matrices on days 5, 7, or 14 (S4 Fig). Analysis of EdU incorporation indicated that the ONPs were still in a proliferative stage (S5 Fig). Representative immunocytochemical images on which these analyses are based are shown in S6 Fig.

### In vivo hESC-derived late-stage ONP transplantation into X-SCID rats

**Fig 4:**
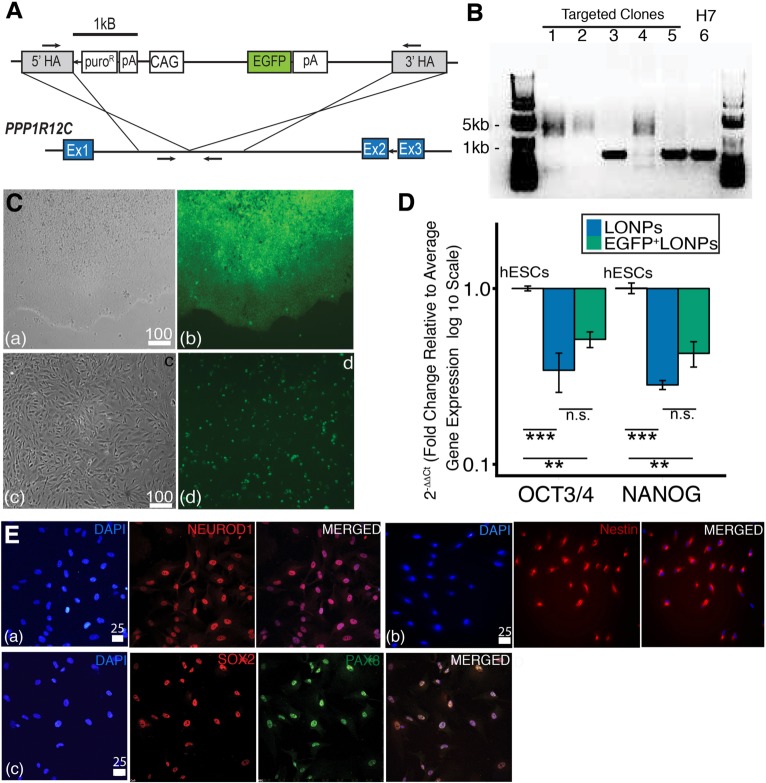
CRISPR-Cas9-modified EGFP^+^ hESCs that differentiated towards late-stage ONP lineage possess late-stage otic neuronal progenitor characteristics. **(A)**: Schematic of AAVS1 safe harbor locus within PPP1R12C gene and targeting vector containing puromycin-resistance cassette (puro^®^), CAG enhancer, and EGFP within 5’ and 3’ homology arms (HA). Arrows indicate PCR primers. **(B)**: EGFP targeting was validated by PCR. H7: H7 human ESCs were used as a control (untargeted clone). (**C**): CRISPR-Cas9 modified EGFP^+^ undifferentiated hESCs in culture. **(Ca)**: phase contrast, **(Cb)**: fluorescence (EGFP^+^) imaging. CRISPR-CAs9 modified EGFP^+^ late-stage ONPs: **(Cc)**: phase contrast, **(Cd)**: fluorescence imaging. Scale bar = 100 μm. (**D**): RT-PCR quantification of OCT3/4 and NANOG expression measurements for H7 hESC, H7 late-stage ONPs (lONPs), and CRISPR-Cas9 modified EGFP^+^ late-stage ONPs. n.s.: not statistically significant, *** *p* < 0.001, and ** *p* < 0.01 by one-way ANOVA with Tukey-Kramer’s post-hoc test. **(E)**: Immunocytochemistry of CRISPR-Cas9 modified EGFP^+^ late-stage ONPs for NEUROD1 **(Ea)**, nestin **(Eb)**, SOX2, and PAX8 **(Ec)**. Scale bar = 25 μm.

In vitro characterization of EGFP^+^-labeled hESCs generated by the CRISPR-Cas9 system with EGFP^+^ constitutively expressed from the AAVS1 safe harbor locus are shown in Fig 4. Fig 4A shows a schematic of the AAVS1 safe harbor locus within the *PPP1R12C* gene and a targeting vector containing a puromycin-resistance cassette (puro^®^), CAG enhancer, and EGFP within 5’ and 3’ homology arms (HA). EGFP targeting was validated by PCR (Fig 4B). The first intron of *PPP1R12C* was PCR amplified with forward and reverse primers complementary to 5' and 3' homology arms, respectively. Correctly edited cells (clone 1, 2 and 4 in Fig 4B) exhibited a shift in amplicon size to 4.7 kb attributable to insertion of the transgene elements (i.e., the exogenous EGFP into the AAVS1 locus) compared to 0.5 kb in targeted, but unedited cells (Clone 3 and 5). Note that Clone 6 was untargeted H7 cells used as a control.

We then characterized EGFP positivity in these cells (Fig 4C, 4D and 4E). Immunocytochemical analysis demonstrates that 55.0% ± 2.2 of EGFP^+^ hESCs retained positivity after differentiation to the late ONP stage (Fig 4C(d)). As expression of EGFP comes at a metabolic cost to the cell, it is anticipated that there will be selective pressure over time during differentiation, reducing the percentage of EGFP^+^ cells (Fig 4C(b) and 4C(d)). RT-qPCR demonstrated significantly decreased mRNA levels of the stem cell markers, NANOG [55] and OCT3/4 [55], suggesting appropriate lineage differentiation of the hESC-derived CRISPR-Cas9-modified EGFP^+^ late-stage ONPs.

The EGFP^+^ labeling did not alter the ability of ONPs to differentiate into late-stage cells in culture (Fig 4E). Representative immunocytochemical analyses of hESC-derived EGFP^+^ late-stage ONPs (H7) demonstrate that the cells have differentiated into neuronal progenitors (NEUROD1 [45], nestin [56], and SOX2 [46]) and also into the late-stage otic neuronal lineage (PAX8 [8,49]). Note that immunocytochemical photomicrographs for PAX8 in Fig 4E are pseudo-colored in green for readers’ convenience. The EGFP^+^ late-state ONPs did not express SOX10 [50], GFAP [52], PAX6 [51], or E-cadherin [53] indicating that the cells have differentiated specifically toward the otic neuronal lineage. These findings are consistent with the late-stage of ONP development described in Fig 2 and also with our previous report [8]. Thus, the CRISPR-Cas9-modified EGFP^+^ hESCs differentiated towards the late-stage ONP lineage, possess late-stage otic neuronal progenitor characteristics (PAX8^+^, SOX2^+^, NEUROD1^+^, and nestin^+^) at the protein level, and show a loss of pluripotency (down-regulation of NANOG and OCT3/4 at the mRNA level).

**Fig 5:**
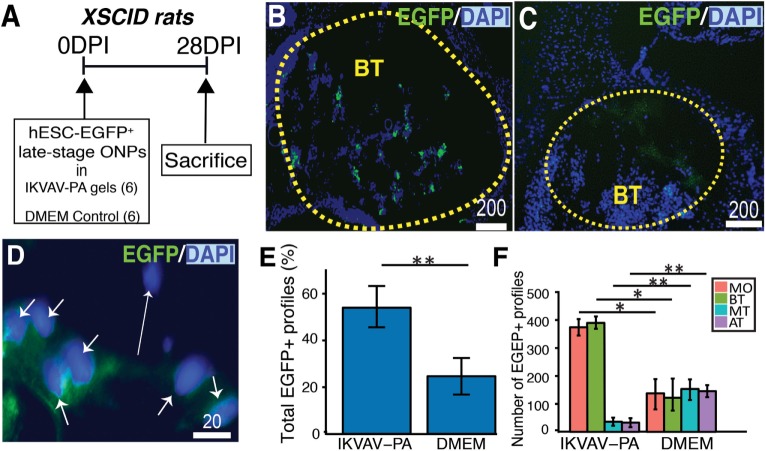
IKVAV-PA gels enhance survival of transplanted EGFP^+^ in the X-SCID rat cochlea. **(A)**: An experimental paradigm. DPI: days post injection. **(B)**: Anti-EGFP antibody labeling of EGFP^+^ cells in the basal turn of the XSCID cochlea. BT: basal turn. Scale bar = 200 μm. **(C)**: Control experiment showing DMEM only injection into the cochlea. Scale bar = 200 μm. **(D)**: Example high-power image of DAPI and EGFP profiles at cellular level used for subsequent quantification. Scale bar = 20 μm. **(E)**: The percentage of EGFP^+^ profiles per section injected with IKVAV-PA gels in and with DMEM (n = 6 for each condition). **(F)**: Comparison of EGFP^+^ profiles in each turn and the modiolus injected with IKVAV-PA gels in and with DMEM (n = 6 for each condition). Abbreviation: MO: modiolus; BT: basal turn; MT: mid turn; and AT: apical turn. * *p* < 0.05, ** *p* < 0.01, and *** *p* < 0.001.

Fig 5A shows our experimental paradigm for transplantation of the EGFP^+^ late-stage ONPs into X-SCID rat cochlea. EGFP^+^ ONPs transplanted with IKVAV-PA gels were found within the cochlea 28 days after transplantation (Fig 5B). DMEM-only injection was performed as a control (Fig 5C). Immunohistochemistry from a sectioned cochlea (Fig 5B) shows expression of EGFP positivity specific to the intracochlear regions of the scala tympani (outlined regions in yellow in Fig 5B). High-power imaging resolved individual cells with (short white arrow) and without (long white arrow) EGFP^+^ staining (Fig 5D); such high-power images facilitated quantitative analyses, summarized in Fig 5E and 5F. Use of IKVAV-PA gels resulted in a significantly greater (*p* < 0.01) number of intracochlear EGFP^+^ profiles (Fig 5E). There also were significantly more EGFP^+^ profiles in the modiolus and basal turn after transplantation with IKVAV-PA gels and significantly less cell migration to higher cochlear turns such as the mid turn and apical turn (* *p* < 0.05, ** *p* < 0.01).

### Ex vivo cadaveric human temporal bone study

**Fig 6:**
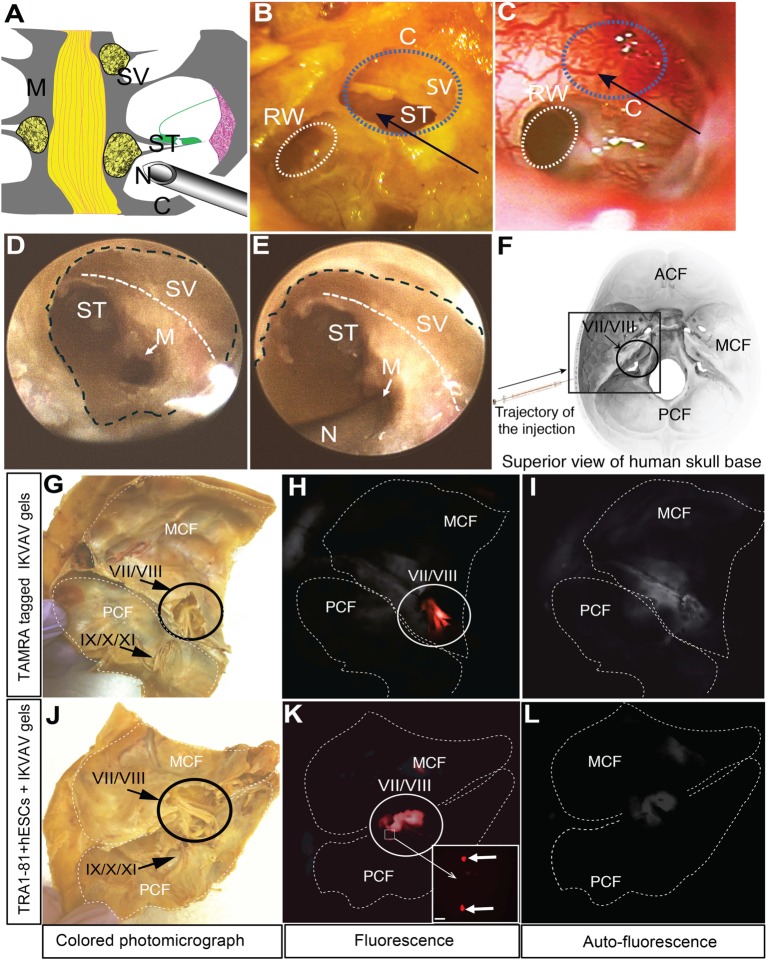
Ex vivo cadaveric human temporal bone study. **(A)**: Schematized midmodiolar cross-section of a mammalian cochlea, showing injection needle approaching the modiolus. **(B-C)**: Photomicrograph of the basal turn of the left cochlea in a cadaveric temporal bone **(B)** and in humans **(C)**. Black arrows indicate direction of injection with IKVAV-PA gels. Note the direction of the fine needle for injection of IKVAV gel containing hESCs. A blue-dotted line shows the cochleostomy site. A white dotted line: round window. **(D and E)**: Endoscopic IKVAV-PA gel injection into human modiolus. View of cochleostomy site using a 16-mm 0° rigid endoscope. A black dashed line marks cochleostomy boundary. A white dashed line indicates presumed plane of the scala vestibuli. **(F)**: Artist’s rendition of the superior view of the human skull base, with black arrow showing the direction of injection of IKVAV gels with hESCs and anatomical landmarks. Black square corresponds to sectioned area in subsequent figures. **(G-L)**: IKVAV-PA gels with hESCs in the IAC in two sets of human cadaveric temporal bones. **(G and J)**: Middle cranial fossa view of the human cadaveric temporal bone before the hESC injection. TAMRA-tagged IKVAV gels **(H)** and corresponding autofluorescence measurement observed in normal temporal bone tissue abutting the IAC **(I)**. TRA-1-81 tagged hESCs with magnified inset **(K)** and corresponding autofluorescence measurement **(L)**. Abbreviations: RW: round window; C: cochleostomy site; ST: scala tympani; SV: scala vestibule; M: modiolus; N: spinal needle; ACF: anterior cranial fossa; MCF: middle cranial fossa; PCF: posterior cranial fossa. Cranial nerves are denoted by Roman numerals.

We then tested the potential clinical feasibility of transmastoid injection of hESCs embedded in IKVAV-PA gels using cadaveric human cochleae. Fig 6 summarizes anatomical landmarks and the presence of IKVAV-PA gel in injected cadaveric human cochleae. A schematic of a coronal section of a human cochlea at the level of mid-modiolus is shown in Fig 6A. Note the trajectory of the needle accessing the modiolus. The same trajectory is illustrated in human cadaveric temporal bone in Fig 6B, which shows the basal turn of the left cochlea, revealed after tympanomastoidectomy in the cadaveric temporal bone. The cochleostomy (C) provided access to the scala tympani (ST) and scala vestibule (SV). For comparison, Fig 6C is an intraoperative image of the basal turn of another left human cochlea from a patient who underwent a mastoidectomy. The black arrows in Fig 6B and 6C indicate a favorable direction for cell injection into the modiolus through the cochleostomy site (blue dotted line).

Based on the requirement for this specific 3-D approach in human temporal bone, we chose a 16-mm 0° rigid micro-endoscope, which provides a favorable angle to the modiolus for the injection of hESCs into the cochlea. It should be noted that a conventional operating microscope would be unable to provide this angle of approach. Using the micro-endoscope, we again performed a cochleostomy on the basal turn of a human cadaveric cochlea. Endoscopic photomicrographs show the injection site at the medial aspect of the scala tympani in Fig 6D (with no needle) and 6E (with the injection needle). Note that the needle trajectory was central through the medial wall of scala tympani to access the modiolus (Fig 6A, 6D and 6E), indicating the successful transcochlear approach to the modiolus and IAC without disruption of the other scalae. Fig 6F, which depicts an artist’s rendition of the human skull base indicating the region examined in subsequent images, provides the trajectory of the injection needle in relation to the human skull base. Note the black arrow for the trajectory of the injection.

Having identified the specific injection trajectory, we then performed injection of IKVAV-PA gels with/without hESCs. Undifferentiated hESCs were used for this proof-of-concept study of our surgical approach as they could be easily identified by detection of the cell surface marker, TRA-1-81. Fig 6G shows the middle cranial fossa of a human cadaveric temporal bone before injection. We sought confirmation that TAMRA-tagged IKVAV-PA was delivered into the IAC of the cadaveric temporal bone through the transmodiolar injection. One hour after injection, endoscopic examination revealed orange fluorescence corresponding to the fluorophore-tagged IKVAV-PA medial to the IAC fundus (Fig 6H). For comparison, autofluorescence of the bone in absence of TAMRA-tagged gel is evident in Fig 6I. Passage of IKVAV-PA gel from the modiolar injection to the inner confines of the IAC was observed within 1–2 minutes. We also visualized the location of the hESCs in the gel delivered to the IAC. Following similar injections in different human cadaveric temporal bones, fluorescent TRA-1-81^+^ hESCs in IKVAV-PA gel were also found overlying the medial aspect of the VII/VIII nerve complex (Fig 6J and 6K). Of note, a moderate amount of autofluorescence was observed in normal temporal bone tissue abutting the IAC using in vitro imaging, and this was used as a control condition to verify positive TRA-1-81 staining of hESCs prior to transmodiolar injection (Fig 6L). Taken together, both TAMRA-tagged IKVAV-PA gels and TRA-1-81 tagged hESCs were successfully delivered into the IAC on the middle cranial fossa side of the temporal bone using transmastoid injection.

### Nanofiber characteristics in vitro and ex vivo after injection of IKVAV-PA Gels

**Fig 7:**
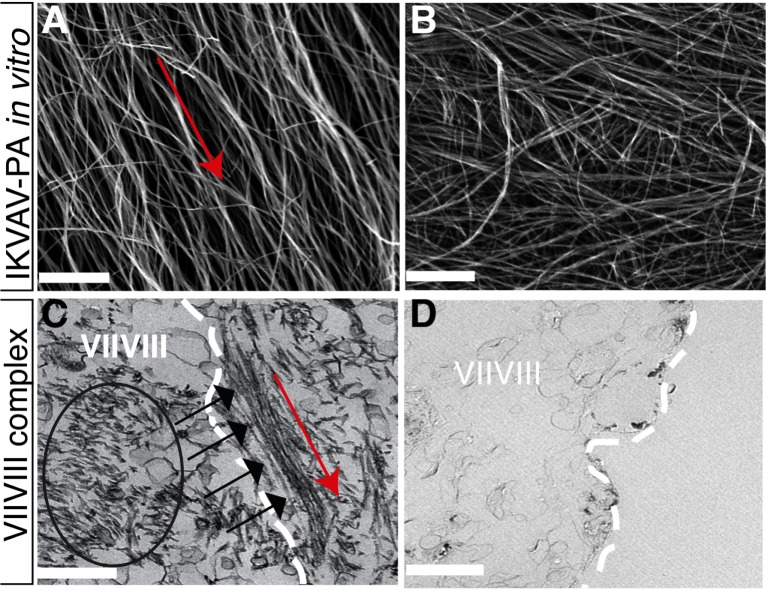
Electron microscopy study of IKVAV-PA gels in vitro and ex vivo human cadaveric tissue. (**A**): SEM image of aligned IKVAV nanofibers in vitro (note predominant orientation in the direction of the shear force, indicated by red arrow). Scale bar = 1 μm. (**B**): Non-aligned IKVAV nanofibers in vitro showing a randomly oriented configuration due to fluid turbulence. Scale bar = 1 μm. (**C**): TEM image of self-assembled IKVAV-PA gel overlying the facial-vestibulocochlear nerve complex (indicated by a black circle and black arrows) following endoscopic transmastoid injection into the modiolus. Note the parallel alignment of self-assembled peptides indicated by the red arrow (11,000× magnification). Scale bar = 1 μm. (**D**): Control nerve specimen with no evidence of peptide scaffold for comparison (1,900× magnification). Scale bar = 1 μm. Note putative epineurium of the facial-vestibulocochlear nerve complex indicated by the white dotted line (C & D). Abbreviations: VIIVIII: facial-vestibulocochlear nerve complex.

IKVAV-PA gel forms nanofibers in vitro that align parallel to an applied shear force when laminar flow is maintained during processing (Fig 7A). However, much of this alignment is lost if laminar flow is not maintained (Fig 7B). We used transmission electron microscopy (TEM) to determine whether fiber alignment is maintained after injection of IKVAV-PA gels into cadaveric human IAC containing the auditory nerve. Examination of the nerve complex following IKVAV-PA injection revealed a parallel alignment of self-assembled nanofibers within the gel that formed upon injection (Fig 7C). These fibers also appeared to orient in the direction of the applied shear force (radially along the longitudinal axis of the nerve), thus mirroring the anatomic arrangement of the VII/VIII nerve complex directed toward the cochlear nucleus. No evidence of similar peptide scaffolds was noted in high-powered images of control nerve tissue (e.g., Fig 7D).

## Discussion

Targeted and stable placement of hESC-derived cells will be essential for a successful cell replacement therapy [57,58]. In the case of hearing, with the exquisite tonotopic organization of the cochlea and its innervation, precise cell targeting may strongly influence the degree of hearing restoration in clinical cases of spiral ganglion cell loss. Although ectopic placement of stem cell-derived SGNs has been successfully demonstrated [4], future progress may depend upon the more controlled delivery of cells along the baso-apical extent of the cochlea to restore proper pitch perception.

Despite the remarkable benefits of cochlear implants (CIs), users frequently note poor speech perception in noisy environments and often find it challenging to appreciate music [2]. This reflects, at least in part, the irreversible retrograde trans-synaptic degeneration of SGNs in this patient population [3]. An increased, “more normal” SGN population could benefit CI designs, allowing for more channels (i.e., frequency resolution), improved sensitivity, and decreased power consumption. Thus next-generation CIs may incorporate targeted cell delivery in tandem with multi-electrode designs to preserve tonotopic nerve stimulation [59]. Further, if hair cell regeneration becomes feasible, successful inner ear function will depend upon the ability of SGNs to effectively transmit signals to the brainstem. We have developed a protocol for the differentiation of cultured hESCs into cells that display the phenotypic characteristics of human SGNs and that preferentially innervate the appropriate central target, the cochlear nucleus, in co-cultures with rat brainstem slices [8]. The present study extends that work by assessing new techniques for promoting differentiation and survival of hESC-derived late-stage ONPs with IVAV-PA gel matrices that promote directional axon growth and cell migration toward desired anatomic inner ear targets. We focused on neuronal differentiation of mid ONPs into late ONPs because late hESC-derived ONPs are the choice of neuronal differentiation stage for in vivo transplantation, as opposed to fully matured in vitro-generated neurons [5,25,60,61] (Please also see a comprehensive review in Needham et al. [62]).

Several factors may underlie low previously reported low survival rates of transmodiolarly injected stem cells [5–7,26], including the relatively inhospitable environment of the bony inner ear, immunorejection by an incompatible host, and inadequate levels of trophic factors supporting cell growth and differentiation [63]. Also, transplantation of single-suspended cells without biomaterial scaffolds such as IKVAV-PA gels that can provide structural and trophic support to neurons leads to apoptosis [64]. IKVAV-PA gels offer a novel means to mitigate these obstacles by providing a more favorable environment for embedded cells. Our results indicate that a greater number of transplanted ONPs remained viable in IKVAV-PA gels as compared to control (DMEM only) media, even after 28 days in vivo. Other 3-D biomaterials for promoting stem cell viability are commercially available (i.e., collagen, laminin, and Matrigel^™^), but they do not allow the same degree of biochemical and biophysical customization as PAs [11,18,65–67]. Furthermore, the self-assembly property of IKVAV-PA allows the material to be injected as liquid into the inner ear where it then gels into an extracellular matrix [11,16–19].

PAs offer advantages beyond those examined here. They can be engineered to present different biologically active molecules to surrounding cells [11,18,30,68]. By adjusting the moiety of the head group during PA synthesis, many small epitopes can theoretically be presented [11]. IKVAV-PA gels have been shown both to decrease stem cell differentiation into astrocytes and to induce copious neurite outgrowth from neurons that far exceed that induced by laminin or isolated IKVAV peptides alone [11]. In our study, IKVAV-PA gels maintained cell viability and promoted ONP differentiation of nearly all transplanted cells. TEM images demonstrated that IKVAV-PA gels formed an aligned self-assembled scaffold over the vestibulocochlear nerve (VCN) complex in the IAC shortly following transmodiolar injection. The liquid crystalline nature of IKVAV-PA enables it to form mechanical guiding frameworks that could direct neurites or new SGNs toward desired neural targets, such as the cochlear nucleus. Next-generation cochlear implants could exploit this feature further by providing IKVAV-based conduits for preferential SGN growth, thereby improving implant performance.

Of interest, IKVAV-PA gels in our study enhanced the differentiation of late ONPs into spiral ganglion-like neurons compared to a control PA gel with a scrambled non-functional epitope (VVIAK), although both gels led to similar cell survival and proliferation. This implies that the survival of the cells is supported by the 3-D matrix itself, whereas the differentiation of the late ONPs is directed by the presence of the laminin epitope incorporated into the PA. This is consistent with previous work showing that the IKVAV epitope leads to neurite extension, specific differentiation toward the neural lineage, and suppression of astrocytic differentiation of ESCs by induction of β1-integrin signaling [11,18,19,21]. Regardless of epitope attachment, 3-D hydrogel scaffolds have been shown to be suitable substrates for cell growth, in many cases preferable to a 2-D substrate [69]; it is therefore unsurprising that cell survival is consistent between the PA gels tested. Our study is unique in that it explores the differentiation and survival of hESCs within the IKVAV-PA gels, as opposed to other work that focused on the extension of the neurites of murine SGN explants to a cochlear implant coated in IKVAV-PA gels [21].

In this work, we observed that IKVAV-PA gels formed in vitro remained intact for at least seven days, the longest time assessed. Although the bio-degradative characteristics of the IKVAV-PA gels in the inner ear remain an open question, a similar injected gel remained within a spinal cord injection site for at least 6 days post-injection [18]. Most recently, Gadolinium tagged PA-hydrogels that were transplanted in mouse muscle were imaged with MRI. The result indicated that the half-life of the PA-hydrogels was 2 weeks. Some of the injected PA-gels were identified even after 4 weeks by inductively coupled plasma-mass spectrometry [70]. Note that the PA-gels used in this study were transplanted into leg skeletal muscle; a tissue that undergoes more mechanical stress than the inner ear. It is therefore reasonable to assume that degradation of PA-gels in the inner ear can be more favorable than the leg skeletal muscle.

Through manipulation of the molecular design of PAs, it is possible to modify the gelation kinetics and mechanical properties [71–73]. In future studies, modulation of the rate of degradation could be assessed to determine if tuning this property impacts stem cell integration in vivo. Further, the degree of inner-ear penetration after IKVAV-PA gel injection can be also manipulated by modifying the PA amino acid sequence, thus changing its viscosity, solidification time, and extent of nanofiber alignment.

Inner ear injections have been well tolerated in vivo in animals. No significant changes between preoperative and 14-day-postoperative auditory brainstem responses were observed after a 5-μL intramodiolar injection of isotonic saline in guinea pigs. Subsequent histologic examinations of the modiolus demonstrated comparable SGN densities between injected and control groups [25]. Although our study used somewhat larger injection volumes, they did not significantly exceed the known volume of the human IAC [74]. IKVAV-PA gels have been proven safe in animal models, with no reported toxicity after injection into the nervous system or other organs [17,19]. However, long-term safety of injection into the inner ear has yet to be established.

We used X-SCID rats to avoid immunorejection of the human cells, facilitating in vivo study of the feasibility of using IKVAV-PA gels. Use of autologous iPSCs for humans would obviate the need for immunosuppression. ONPs transplanted by transmodiolar injection exhibited higher survival rates with IKVAV-PA gels compared to DMEM, consistent with our in vitro results. Further, the goal of transmodiolar injection is to deliver ONPs to the modiolus, and a larger proportion of surviving ONPs was found in the modiolus with IKVAV-PA gels. This suggests that the transition of IKVAV-PA from liquid to gel limited undesirable flushing of cells away from the injection site. Notably, with DMEM-based injection used a controlled condition, a larger proportion of ONPs was located in the mid-apical cochlear turns, suggesting that ONPs were flushed away from the modiolar injection site. Thus, IKVAV-PA gels not only increased survival of transplanted cells, but also facilitated more precise and efficient delivery of the cells to clinical sites of interest.

The successful injection of IKVAV matrices into human cadaveric temporal bone with a transmodiolar approach has positive implications for safer clinical approaches for stem cell delivery. Transmodiolar delivery of ONPs would likely have far less comorbidity than traditional middle cranial fossa or suboccipital IAC approaches. Middle cranial fossa approaches typically require extended retraction of the temporal lobes, increasing the risks of seizure, intracranial bleeding, aphasia, and iatrogenic CSF leakage [75]. Similarly, the suboccipital approach can potentially cause severe headache and CSF leakage postoperatively [76]. In a rat model using a suboccipital approach, a fascial patch was used to repair the underlying dura, a step potentially increasing risk of intracranial complications [62]. These more invasive techniques can be avoided with the use of a self-assembling peptide that can be injected as a liquid, effectively removing the need to place biomaterials with a higher viscosity directly on the IAC portion of the SGNs. Furthermore, our transmodiolar injection technique would obviate the need for suboccipital craniotomy, a riskier approach proposed as a means to circumvent neurite growth inhibition at the ORZ [23,63,77]. Our results also indicate that rigid endoscopes may be helpful adjuncts for the surgeon to visually confirm the correct anatomic injection technique and avoid collateral damage.

## Conclusions

In this study, we used IKVAV-PA gels to create a niche that supported otic neuronal differentiation and survival in vitro and in vivo. Culture of human hESC-derived ONPs in IKVAV-PA hydrogels did not alter proliferation or viability of the cells, but improved neuronal differentiation and neurite outgrowth. Injection of IKVAV-PA gels along with hESC-derived ONPs into the modiolus of X-SCID rats and cadaveric human temporal bone demonstrated the technical feasibility of a clinical transmastoid approach. Thus, combining stem cell transplantation with the injection of self-assembling PAs to create a supportive tissue microenvironment may advance the goal of clinical inner ear regeneration.

## Supporting information

S1 Fig Typical images collected for secondary antibody only control samples. Samples were treated identically to experimental immunocytochemistry conditions with the omission of primary antibody during overnight incubation. **(A)**: Fluorescence images for a secondary antibody control experiment using goat anti-rabbit Alexa Fluor 488 (G-anti-RB-488) and donkey anti-mouse Alexa Fluor 647 (D-anti-M-647). Scale bar: 100 μm. **(B)**: Fluorescence images for a secondary antibody control experiment using donkey anti-goat Alexa Fluor 488 (D-anti-G-488) and donkey anti-mouse Alexa Fluor 647 (D-anti-M-647).
(EPS)

S2 Fig RT-PCR quantification of GAPDH expression measurements for H7 hESCs, H7 late-stage ONPs (L-ONPs), and H7 EGFP^+^-ONPs. (EPS)

S3 Fig Rheological measurements of PA gels. **(A)**: Storage modulus of E2 PA, IKVAV PA and VVIAK PA measured in pascals. **(B)**: Strain sweep of E2 filler PA (a), IKVAV-PA (b), and VVIAK-PA (c) showing G’ and G” with increasing percent shear strain. **(C)**: Time sweep of all materials showing storage modulus, G’, and loss modulus, G” with increasing time exposed to physiological ion gelling solution. **(D)**: G”/G’ or tan(δ), vs. angular frequency in rad/s. *p <0.05, **p <0.01, and ***p <0.001. Fisher’s Least Significant Difference test was performed (n = 3).
(EPS)

S4 Fig Quantification of Live/Dead cell viability assay on S/R/F/E/I-treated hESC-derived mid-stage ONPs. No significant difference was noted among the three matrices.
(EPS)

S5 Fig Quantification of EdU-positive cells on S/R/F/E/I-treated hESC-derived mid-stage ONPs. (EPS)

S6 Fig A representative image of Live/Dead assay. **(A)**: Human hESC-derived mid-stage ONPs that were embedded in IKVAV-PA gels stained with calcein at DIV5. **(B)**: Human ESC-derived mid-stage ONPs that were embedded in IKVAV-PA gels stained with calcein at DIV7. Both images (A and B) demonstrate green fluorescence corresponding to viable cell populations. Refracted light seen during in vitro imaging was attributed to the depth of the 3-D gel along with a nonplanar distribution of cells. **(C)**: hESC-derived mid-stage ONPs that were embedded in IKVAV-PA gels stained merged image at DIV14 showing numerous viable cells (green) with minimal apoptosis (red). Magnified portion of image is shown in a white square with two neurites noted by white arrows. Scale bar: 20 μm.
(EPS)

S1 Supporting Information Supplemental materials and methods. (DOCX)

S2 Supporting Information Rheological measurements of PA-hydrogels. (DOCX)

S3 Supporting Information Addendum to discussion. (DOCX)
