# Supplementary material for: Creating a stem cell niche in the inner ear using self-assembling peptide amphiphiles
Source: PLoS One. 2017 Dec 28;12(12):e0190150. doi: 10.1371/journal.pone.0190150 (PMC5746215; doi:10.1371/journal.pone.0190150)
Supplement: S1 Supporting Information — (DOCX) [file pone.0190150.s007.docx]

**Supporting Information (Supplemental Materials and Methods)**

**Media formulations**

Note that All percentages are computed as vol/vol.

**Human ESC Culture Medium**

75% DMEM-F12 medium, 20% Knockout Serum Replacement, 1 mM L-Glutamine, 4 ng/mL beta-FGF, 1% Non-Essential Amino Acids (NEAA), 100 µM beta-mercaptoethanol (beta-ME) (all reagents for CDM were from Life Technologies, Carlsbad, California, USA).

**N2B27-based Chemically Defined Medium (N2B27-CDM)**

1% N2 supplement, 2% B27 supplement, 2 mM glutamine, 100 μM beta-mercaptoethanol in a 1:1 ratio of Dulbecco’s modified eagle medium (DMEM)/F12 and Neurobasal medium (all reagents for CDM were from Life Technologies, Carlsbad, CA, USA).

**ONP maintenance medium (ONPMM)**

CDM, 20 ng mL^-1^ EGF (3.2 nM), 10 ng mL^-1^ FGF2 (1.2 nM), and 50 ng mL^-1^ IGF-1 (6.6 nM) (All human recombinant reagents were from R&D Systems, Minneapolis, Minnesota, USA).

**Neuronal Induction medium (NIM)**

Neurobasal medium (ThermoFisher, Waltham, Massachusetts, USA) or BrainPhys™ (Stem Cell Technologies, Cambridge, MA, USA), 1% N2, 2% B27, 1 mM Glutamax™ (ThermoFisher, Waltham, Massachusetts, USA). Note that All percentages are computed as vol/vol.

**Synthesis of IKVAV epitope**

The laminin derived epitope, IKVAV (isoleucine-lysine-valine-alanine-valine) peptide amphiphile (PA) and non-bioactive E2 PA were synthesized by fluorenylmethoxycarbonyl (Fmoc) chemistry using solid-phase peptide synthesis and purified using preparative high-performance liquid chromatography (HPLC) with H_2_O and acetonitrile mobile phase. Pre-derivatized Wang resins (EMD Millipore, Kankakee, Illinois) and Fmoc-protected amino acids (Anaspec Inc, Fremont, CA) were used for solid phase synthesis. Palmitic acid groups were added to the synthesized peptide segments, after which PAs were deprotected and cleaved from the resin using a solution of 95% trifluoroacetic acid, 2.5% triisopropyl silane, and 2.5% water for three hours. The resulting solution was transferred to a round bottom flask, and the resin was rinsed with DCM. Liquid was removed by rotary evaporation to yield solid PA powder, which was rinsed with cold diethyl ether and poured into a fritted filter. The solution was rinsed on the filter again and dried.

Crude PA powder was dissolved in water with 0.5 M ammonium hydroxide, pH adjusted to 9-10 using additional ammonium hydroxide as needed. The resuspension was passed through a 0.22 μm filter and purified on a Phenomenex C18 Gemini NX column by reverse phase HPLC running a mobile phase gradient of 98% water with 2% acetonitrile (HPLC grade, Mallinkrodt, St. Louis, Missouri, USA) to 100% acetonitrile. To aid solubility of the PA, 0.1% NH­_4_OH was added to all mobile phases. HPLC fractions were checked for correct mass of compound using electrospray ionization mass spectroscopy and concentrated under reduced pressure to remove residual acetonitrile. The resulting PA concentrated was frozen and lyophilized in a FreeZone6 system (Labconco, Kansas City, Missouri, USA) to obtain a PA powder.

PA powder was dissolved in a solution containing 140 mM NaCl and 3 mM KCl (Sigma Aldrich, St. Louis, MO, USA). All PA solutions were then adjusted to approximately pH 7.4 using 1M NaOH. Final NaOH concentrations were approximately 17 mM. E2 (C16V2A2E2) and IKVAV (C16V2A2E4GIKVAV) solutions were mixed at a 3:1 ratio to obtain a final concentration of 0.83 wt% E2 PA and 0.625 wt% IKVAV PA. Afterwards, PAs were annealed at 80 ºC for 30 minutes and slowly cooled at a rate of 1ºC per minute until room temperature was reached. This process promotes the formation of long fibers required for the formation of robust nano-fibrous networks.

**Human ESC cultures**

The human ESC lines H1 (WA-01, XY, passage 25-30), H7 (WA-07, XX, passages 25–35), and H9 (WA-09, XX, passage 25-35) were grown in adherent cultures on gamma-irradiated mouse embryonic fibroblasts (MEFs) in hESC culture medium. Alternatively, the hESCs were cultured in feeder-independent conditions on Geltrex™ in mTeSR™1 medium. Standard plating density of freshly irradiated MEFs was 0.75 x 10^5^ cells mL^-1^ at 2.5 mL per well in a 6-well plate. The undifferentiated hESCs were passaged using collagenase IV (Life Technologies, Carlsbad, California, USA) every 5-7 days. Prior to splitting, all undifferentiated colonies were assayed for morphology, and all imperfect colonies were manually removed.

**Immunocytochemistry**

CDM was aspirated and embedded cells were washed with PBS containing 1 mM CaCl_2_. The cells embedded in gels were fixed with 4% paraformaldehyde for 20 minutes at room temperature by submerging the entire PA-gel in fixative. The cells were permeabilized using 0.25 % Triton X-100 (Sigma-Aldrich, St. Louis, Missouri) on day 15 in PBS for 10 minutes at room temperature and washed with PBS three times for 5 minutes each or three times for 20 minutes each for PA gels. After blocking in 5% BSA (Sigma-Aldrich, St. Louis, Missouri, USA) for 1 hour at room temperature, primary antibodies were added overnight at 4°C. PA gels were permeabilized and blocked simultaneously in a PBS solution containing 5% FBS and 0.1% Triton X-100. For secondary antibody only controls, primary antibodies were omitted for this step. Subsequently, cells were incubated for 45 minutes with fluorescence-labeled secondary antibodies at RT, 2 hours for PA gels. The coverslips were washed with PBST (0.5% Tween-20 in PBS) and mounted using ProLong® Gold Antifade Mountant with DAPI (Life Technologies, Carlsbad, California, USA). PA gels instead were incubated in 300 nM DAPI for five minutes. Primary antibodies used were as follows: NANOG (anti-goat, 1:500, Thermo Fisher Scientific, Waltham, MA, Cat#: PA5-18678), PAX2 (anti-rabbit, 1:500, Abcam, Cambridge, MA, USA, Cat#: AB79389), PAX6 (anti-mouse, 1:500, Sigma-Aldrich, St. Louis, MO, USA, Cat#: AB2237 or anti-rabbit, 1:200, Chemicon International, Temecula, CA, USA, Cat#: AB5409), PAX8 (anti-goat, 1:100, Abcam, Cambridge, MA, USA, Cat#: AB13611), SOX2 (anti-mouse, 1:100, Abcam, Cambridge, MA, USA, Cat#: AB75485), SOX10 (anti-mouse, 1:500, Sigma-Aldrich, St. Louis, MO, USA , Cat#: SAB1402361), GATA3 (mouse, 1:100, R&D Systems, Minneapolis, MN, USA, Cat#: MAB6330), nestin (anti-mouse, 1:100, EMD Millipore, Kankakee, IL, USA, Cat#: MAB5326 or anti-rabbit, Abcam, Cambridge, MA, USA, Cat#: AB105389), NEUROD1 (anti-mouse, 1:100, Abcam, Cambridge, MA, USA, Cat#: AB60704), GFAP (anti-goat, 1:200, Santa Cruz Biotechnology, Dallas, TX, Cat#: SC6170 or anti-mouse, 1:1000, Sigma Aldrich, St. Louis, MO, USA, Cat#: G3893), E-Cadherin (anti-mouse, 1:100, BD Bioscience, San Jose, CA, USA, Cat#: 610181), and β-III Tubulin (anti-mouse, 1:100, Sigma-Aldrich, St. Louis, MO, USA, Cat#: T8660-1000L). Secondary antibodies used were donkey anti-rabbit Alexa Fluor 594 (6 μg/mL, Cat#A21207), donkey anti-mouse Alexa Fluor 647 (4 μg/mL, Cat#A31571), donkey anti-goat Alexa Fluor 488 (6 μg/mL, Cat#A11055), goat anti-rabbit Alexa Fluor 488 (6 μg/mL, Cat#A11034), and donkey anti-mouse Alexa Fluor 488 (6 μg/mL, Cat#A21202) (Life Technologies, Carlsbad, California, USA).  **Image acquisition and quantification of cells**

The level of fluorescence in a given region (e.g., nucleus) was determined using the corrected total cell fluorescence (CTCF) based on the following formula:

***CTCF = Integrated Density – (Area of selected cell x Mean fluorescence of background readings)*** [1]***.*** DAPI-stained cells were counted using the ITCN (image-based tool for counting nuclei) plugin for ImageJ developed by Thomas Kuo and Jiyun Byun at the Center for Bio-image Informatics at the University of California Santa Barbara [2]. Images were converted to 8-bit grey scale and inverted before using ITCN. Cell detection was performed detecting dark peaks with the following parameters: cell width = 7, minimum distance = 7, threshold = 2, mask image: use selected ROI. The number of positively labeled cells was counted in 10 random selected fields and was expressed as a percentage of the total number of cells counted. The total number of cells was quantified by DAPI nuclear staining.

**Live/dead cell viability assay**

In vitro cell viability was assessed using a LIVE/DEAD viability/cytotoxicity kit (Life Technologies, Carlsbad, California). Using ethidium bromide (Ex/Em 517/617 nm) and calcein (Ex/Em 494/517 nm) as markers for abnormal cell membrane permeability and intracellular esterase activity respectively, mid-ONPs treated with S/R/F/E/I in separate wells were evaluated at days 5, 7, and 14. Following permeabilization with 0.1 % Triton X-100, calcein (2 µM) and ethidium bromide (6 µM) were added for 15 minutes prior to microscopic imaging.

**EdU cell proliferation assay**

Click-iT® EdU DNA incorporation assays (Invitrogen, Carlsbad, California, USA) were performed to confirm in vitro cell proliferation. The mid ONPs treated with S/R/E/F/I were fixed at day 5 using 4 % paraformaldehyde followed by permeabilization with 0.5 % Triton X-100 in PBS. A high concentration (40 µM) of EdU reagent incubated for 48 hours was used given our experience noting an increased diffusion time through plated IKVAV-PA gel. In vitro fluorescent imaging was performed using either a Zeiss Axiovert 200M or Molecular Devices ImageXpress Micro microscope at 20x magnification.

**Human ESC-derived ONPs transplantation into the XSCID rat cochlea**

XSCID rats, F344-Il2rg^em7kyo^ were generated by Dr. Tomoji Mashimo at Graduate School of Medicine, Kyoto University Institute of Laboratory Animals and transferred to Northwestern University for further use [3]. Rats were housed in specific pathogen free (SPF) conditions. The Institution Animal Care and Use Committee at Northwestern University Feinberg School of Medicine approved the experimental protocol for this project (IACUC Protocol number: IS00000379), which met National Institutes of Health guidelines for animal care and use. Twelve X-SCID rats were of each sex between six to eight weeks of age were used as stem cell transplant recipients. Two hours prior to incision, preoperative antibiotics, 5 mg kg^-1^ Baytril (Bayer HealthCare, Shawnee Mission, Kansas, USA), was administered subcutaneously to prevent perioperative infection. A weight-appropriate dose of analgesic medication, meloxicam (1 mg kg^-1^) or buprenorphine SR (0.5 mg kg^-1^) was given. Atropine (0.03 mg kg^-1^) was also given to reduce secretion in the airway. Under 2-3% isoflurane, 70% nitrous oxide, and 30% oxygen, the anesthetic plane was tested with pedal withdrawal reflex (toe pinch) using fingernails pinch web of skin between animal's toe. When the limb was not withdrawn and there were no muscle twitches, the animal was then placed on an area separated from where the surgery was conducted and hair was removed from skin around the surgical site. A dedicated area for the operation was first disinfected with 85% isopropyl alcohol with more than 15 minutes of contact time on the surface. A circulating water blanket was placed on the operating table, as Northwestern University IACUC policy 9.120.0 dictates that electric heating pads must never be used as heat support. NaCl saline solution (50 mL kg^-1^) at 37°C was given subcutaneously to help ensure adequate hydration during the procedure. At this time, an ophthalmic ointment was applied in order to prevent corneal drying and corneal abrasions. If the surgery proceeded longer than 2 hours, medications were re-administered as necessary. The animal was placed on the sterile drape and a rectal probe was placed into the rectum to monitor core temperature throughout the procedure. Also, an oxygen saturation monitor (with sensor across the animal's toe) to monitor SaO_2_ throughout the procedure. All surgical instruments were sterilized (steam under pressure) prior to the surgery. The animal was prepped with swabbing with Betadine (Purdue Products, Stamford, Connecticut) followed by applying 70% alcohol. This procedure was repeated 3-5 times. Sterility of instruments was maintained during procedures by using “tip-only” technique and between surgeries with a glass-bead sterilizer.

Detailed description of surgical procedures can be found in Northwestern University IACUC protocol policy #9.109.0. Briefly, prior to surgery, the animal was inspected to ensure that it was free of signs of ear infection using the operative microscope. A small incision (from midline, through bregma, and then laterally toward the jugular process) was made to expose the left posterior aspect of the skull. After dissecting the sternocleidomastoid muscle, the mastoid bulla was identified. The bulla was entered using a small dental drill followed by the exposure of the round window niche. A 33-gauge beveled NanoFil needle (World Precision Instruments, Sarasota, Florida, USA) was used to create a small fenestra in the bony wall of the basal turn slightly lateral to the round window niche, allowing access to the modiolus. A suspension of cultured hESC-derived late ONPs (2 x 10^4^ cells) of IKVAV solution or culture medium was used for an injection using a 33-gauge blunt NanoFil needle. The injection was performed with a setup that consists of Magnetic base for 12 mm Clamp, a Micromanipulator M3301R, UltraMicroPump (UMP3-1), a 10-μL Hamilton syringe (World Precision Instruments, Sarasota, Florida, USA).

After the injection, the defect of the mastoid bone was cemented closed using Durelon™ Carboxylate Luting Cement (3M EPSE Dental, St Paul, Minnesota, USA). After confirming that the dental cement was firm and solid, a two-layer closure was made. A coated 3-0 Polyglacten 910, Vicryl™ (Ethicon, Somerville, New Jersey, USA), was used for muscle-layer closure and a 3-0 polybutester, Novafil™ (Coviden, Dublin, Republic of Ireland) was used for skin closure. Stitches were performed in an interrupted fashion so as to avoid wound dehiscence. At the conclusion of the surgery, the rat was once again given Buprenorphine SR or meloxicam subcutaneously for postoperative pain. The animal was allowed to recover in its cage positioned over a heating pad. Post-surgical discomfort was treated with Meloxicam (1 mg/kg) or buprenorphine SR (0.5 mg kg^-1^) subcutaneously.

Four weeks after the transplantation surgery, the animal was deeply anesthetized with isoflurane and 30 mL of PBS and transcardially perfused with 0.9% saline at 37°C followed by 4% paraformaldehyde in 0.1 M phosphate buffer (pH 7.4) at 4°C. The temporal bone containing the cochlea was excised and kept in the same fixative before being transferred to 0.1 M PBS. After rinse with PBS, the temporal bone was thinned with a diamond burr to create a small opening along the striae vascularis to facilitate fluid uptake into the scalae. The temporal bone was then decalcified by immersing it into a 0.1 M ethylenediamine-tetraacetic acid (EDTA) solution for 10 days with gentle agitation. Cochleae were then immersed in 30% sucrose solution overnight and then embedded into optimum cutting temperature embedding compound (OCT Embedding Compound, Triangle Biomedical Sciences, Durham, North Carolina, USA). Transverse cryostat sections of the cochlea were cut at 10 μm parallel to a mid-modiolar plane and images of every fourth section throughout the cochlea were collected. Immunohistochemical staining was performed to detect hESCs with EGFP using an anti GFP antibody (1:500, rabbit polyclonal to GFP antibody, Abcam, Cambridge, Massachusetts, USA). The photomicrographs shown in Figure 5B, 5C, and 5D are pseudo-colored in green for reader’s convenience.

**Rheological measurements of PA-hydrogels.**

PA materials were prepared using methods described above. An Anton Paar MCR302 Rheometer with a 25mm cone plate was used for all rheological studies. 150µL of PA liquid was placed on the sample stage and 30µL of 150mM CaCl_2_ solution (final concentration 25mM) was placed on the sample plunger positioned above the material. The instrument stage was set to 37°C. The plunger was lowered to the measuring position and a humidity collar was added to prevent sample evaporation. The sample was equilibrated for 30 minutes with a constant angular frequency of 10 [rad/s] and 0.1% strain and the storage and loss modulus were recorded over time. The angular frequency was then incremented from 100 rad/s to 1 rad/s over 21 points and the storage and loss modulus were recorded. The tan(δ) was obtained from the ratio of G’’/G’ during this interval. Lastly, the percent strain was incremented from 0.1 to 100% over 31 points and the storage and loss modulus were reported.

**Quantification of the surviving EGFP^+^ hESC-derived ONPs**

The five most central modiolar sections were chosen, with each separated by 30 μm, defining a reference space of 200 μm. The most central modiolar sections for all groups were then stained. Profile counts were generated from a microscopic image digitized using ImageJ software. The total profile number was calculated by counting profiles of the EGFP^+^ cells on these five most central modiolar sections. Only cells with clearly visible EGFP fluorescence (or with the confirmation of GFP antibody immunostaining) were counted as surviving ONPs. The profile number obtained from each of the five sections was multiplied by 4 because every fourth section was kept for analysis. The total profile number of the EGFP-positive cells was determined for each of the five sections of each animal in four anatomic subdivisions of the cochlea: basal turn, mid turn, apical turn, and modiolus [4].

**Electron microscopy (TEM)**

Portions of the VII/VIII cranial nerve complex following IKVAV-PA injection were harvested and sectioned into 1mm^2^ tissue samples. Samples of the VII/VIII cranial nerve complex without nanogel coating were also harvested as control specimens. Tissue was stored in 4% paraformaldehyde with 1 mM CaCl_2_ at 4^°^C prior to dehydration in serial EtOH washes. Specimens were then post-fixed with 2% osmium tetroxide prior to en bloc staining with 3% uranyl acetate. These samples were subsequently embedded in an epoxy resin (EMbed 812, Electron Microscopy Sciences, Hatfield, Pennsylvania, USA) and thick sectioned using a Leica UC6 Ultramicrotome. Final specimens were stained with uranyl acetate and lead citrate followed by 70 nm thin sectioning. Images were obtained using a FEI Tecnai Spirit G2 apparatus (Penn State Huck Institutes of the Life Sciences, University Park, PA, USA).

**Scanning electron microscopy (SEM)**

Aligned IKVAV gels were formed by pipetting a PA solution through a gelling solution composed of 150 mM NaCl, 3 mM KCl, and 25 mM CaCl_2_. Unaligned gels (mimicking VVIAK-PA) were formed by spreading a PA solution on a positively charged glass coverslip and then applying a gelling solution over the initial PA matrix. The gels were dehydrated in graded (15- 100%) EtOH washes and then critical point dried (Tousimis SAMDRI-795, Rockville, Maryland, USA). The dried samples were coated with 5 nm of osmium using an osmium plasma coater (Structure Probe Inc., West Chester, Pennsylvania) and imaged with a Hitachi S-4800 field emission scanning electron microscope.

**References**

1. Burgess A, Vigneron S, Brioudes E, Labbé J-C, Lorca T, Castro A. Loss of human Greatwall results in G2 arrest and multiple mitotic defects due to deregulation of the cyclin B-Cdc2/PP2A balance. Proc Natl Acad Sci U S A. 2010;107: 12564–12569. doi:10.1073/pnas.0914191107

2. Byun J, Verardo MR, Sumengen B, Lewis GP, Manjunath BS, Fisher SK. Automated tool for the detection of cell nuclei in digital microscopic images : Application to retinal images. Mol Vis. 2006;12: 949–960.

3. Mashimo T, Takizawa A, Kobayashi J, Kunihiro Y, Yoshimi K, Ishida S, et al. Generation and characterization of severe combined immunodeficiency rats. Cell Rep. The Authors; 2012;2: 685–694. doi:10.1016/j.celrep.2012.08.009
