# Supplementary material for: Creating a stem cell niche in the inner ear using self-assembling peptide amphiphiles
Source: PLoS One. 2017 Dec 28;12(12):e0190150. doi: 10.1371/journal.pone.0190150 (PMC5746215; doi:10.1371/journal.pone.0190150)
Supplement: S2 Supporting Information — (DOCX) [file pone.0190150.s008.docx]

**Supporting Information (Rheological measurements of PA-hydrogels)**

We have performed a rheological study of the IKVAV, VVIAK and E2 PA hydrogels after gelation (S3 Fig), and we found that they had storage moduli of 7,042 Pa, 8,024 Pa, and 1,510 Pa, respectively (S3 Fig A). The difference in storage modulus between IKVAV and VVIAK PA gels was not statistically significant. Most importantly, the frequency sweep showed G’> G’’ in all cases at all frequencies indicating the material is in a gelled state. In all materials, tan(δ) or (G’’/G’) is always less than 1; meaning that the sample is more elastic than viscous, in other words, it is an “elastic solid” (S3 Fig D).

One of the important biophysical properties of PA-gels (i.e., IKVAV PA-gels in this study) is the ability to transition from a liquid to a gel after injection in vivo as it is technically not feasible to place gelling material in the inner ear. We tried to measure the sol-gel transition, but the material gels instantly upon contact with physiological ions as seen in S3 Fig C where G’>G’’ at the first timepoint, which is 5 seconds. Our rheological instrumentation is not equipped to observe this rapid transition. It would be possible to record rheological data of the material without gelling solution, but this will not measure the transition. Furthermore, un-gelled data has been reported in multiple previous papers and this specific analysis is outside the scope of this paper. IKVAV PA gels have been shown to form gels after injection in vivo in at least five manuscripts that have been previously published [1–5].

**References**

1. Silva GA, Czeisler C, Niece KL, Beniash E, Harrington DA, Kessler JA, et al. Selective differentiation of neural progenitor cells by High-epitope density nanofibers. Science. 2004;303: 1352–1355. doi:10.1126/science.1093783

2. Tysseling VM, Sahni V, Niece KL, Birch D, Czeisler C, Fehlings MG, et al. Self-Assembling Nanofibers Inhibit Glial Scar Formation and Promote Axon Elongation after Spinal Cord Injury. J Neurosci. 2008;28: 3814–3823. doi:10.1523/JNEUROSCI.0143-08.2008

3. Tysseling VM, Sahni V, Pashuck ET, Birch D, Hebert A, Czeisler C, et al. Self-assembling peptide amphiphile promotes plasticity of serotonergic fibers following spinal cord injury. 2010;88: 3161–3170. doi:10.1002/jnr.22472

4. Berns EJ, Sur S, Pan L, Goldberger JE, Suresh S, Zhang S, et al. Aligned neurite outgrowth and directed cell migration in self-assembled monodomain gels. Biomaterials. Elsevier Ltd; 2014;35: 185–195. doi:10.1016/j.biomaterials.2013.09.077

5. Pan L, North HA, Sahni V, Jeong SJ, Mcguire TL, Berns EJ, et al. β1-Integrin and integrin linked kinase regulate astrocytic differentiation of neural stem cells. PLoS One. 2014;9: e104335. doi:10.1371/journal.pone.0104335
