# Supplementary material for: Creating a stem cell niche in the inner ear using self-assembling peptide amphiphiles
Source: PLoS One. 2017 Dec 28;12(12):e0190150. doi: 10.1371/journal.pone.0190150 (PMC5746215; doi:10.1371/journal.pone.0190150)
Supplement: S3 Supporting Information — (DOCX) [file pone.0190150.s009.docx]

**Supporting Information (Addendum to Discussion)**

Inflammatory reaction and a foreign body immune response of IKVAV-PA gels to host tissues were not assessed in this study. Using similar PA-hydrogels (C16GSH-PA gels) [1], inflammation was assessed at post-implanted day 3 and 10 and no sign of inflammation was previously reported. In unpublished work [unpublished work, Reza Maotalleb, Eric J. Berns, Piyush Patel, Julie Gold, Samuel I. Stupp, H. Georg Kuhn, submitted for publication], a similar gel with a different epitope and at a lower concentration was injected into brain. The neuroinflammatory response was assessed using immunofluorescent staining of reactive astrocytes with GFAP and microglia with Iba1. In our future studies, inflammatory reaction of IKVAV-PA gels and a potential foreign body immune response could be assessed by detecting evidence of multinuclear giant cells or a fibrous capsule at the border between host tissue and the transplanted IKVAV-PA gels in the cochlea.

**Reference**

1. Black KA, Lin BF, Wonder EA, Desai SS, Chung EJ, Ulery BD, et al. Biocompatibility and Characterization of a Peptide Amphiphile Hydrogel for Applications in Peripheral Nerve Regeneration. Tissue Eng Part A. 2015;21: 1333–1342. doi:10.1089/ten.tea.2014.0297
